# An Unrecognized Cause of Elevated Procalcitonin Level

**DOI:** 10.7759/cureus.39475

**Published:** 2023-05-25

**Authors:** Ghadah Thiab, Anthony Workman, Imran Khawaja

**Affiliations:** 1 Internal Medicine, Marshall University Joan C. Edwards School of Medicine, Huntington, USA; 2 Pulmonary Medicine, Marshall University Joan C. Edwards School of Medicine, Huntington, USA

**Keywords:** high anion gap metabolic acidosis, procal, diabetic ketoacidosis (dka), elevated procalcitonin in dka, diabetic ketoacidosis and procalcitonin

## Abstract

Diabetic Ketoacidosis (DKA) is a preventable yet serious complication of diabetes that is commonly associated with type 1 diabetes but can also occur in other forms of diabetes, including type 2. Infection is a primary cause of DKA and can lead to elevated levels of procalcitonin (PCT), which is a biomarker used to differentiate bacterial infections from non-infectious inflammation. However, some cases of DKA have shown increased PCT levels even in the absence of bacterial infection, and the underlying mechanism of this observation is not fully understood. To gain a better understanding of how non-infectious inflammation affects PCT levels, further research is needed.

While PCT is a helpful biomarker, it should be interpreted in the context of the patient's overall clinical picture, including signs and symptoms of infection or inflammation and underlying medical conditions that may be contributing to their presentation. A systematic approach to evaluating and managing patients with DKA can minimize the risk of unnecessary antibiotic use and ensure optimal treatment.

We present a 31-year-old male who was admitted to the ICU with DKA, and further investigations revealed elevated PCT levels. Despite conducting several studies and cultures, including blood and urine, no infections were detected. The patient was treated for DKA with hydration and insulin administration without the use of antibiotics, and his PCT levels subsequently decreased.

## Introduction

Diabetic Ketoacidosis (DKA) is a serious but preventable complication of diabetes. It is more commonly associated with type 1 diabetes; it can also occur in patients with type 2 diabetes or other forms of diabetes [1]. It can be triggered by a variety of factors, including insulin omission, new diagnoses of diabetes, infections, myocardial infarction, thyrotoxicosis, and the use of certain medications such as corticosteroids or antipsychotics [2].

Infection is one of the leading causes of DKA and can cause elevated levels of acute phase reactants, including procalcitonin (PCT) [3]. PCT is a biomarker that is commonly used to differentiate bacterial infections from non-infectious causes of inflammation [4]. However, elevated PCT levels have been observed in some cases of DKA, even in the absence of infection [5,6]. While the exact mechanism behind the elevation of PCT in non-infectious DKA cases is not fully understood, it is known that inflammation plays a role in the pathophysiology of DKA. DKA is characterized by excess ketone bodies in the blood, leading to acidosis and subsequent inflammation. It is possible that this inflammation may trigger the release of PCT from neuroendocrine cells in the lungs and intestine, although further studies are needed to confirm this. This will lead to the wise and cautious use of antibiotics in this patient population, and the careful order of workup will add up to the cost of treatment.

## Case presentation

A 31-year-old Caucasian man presented to an outside facility with abdominal pain, nausea, and vomiting that developed a few hours before admission. The patient had been diagnosed with Type 1 Diabetes at the age of 19 and has been using an insulin pump with a basal rate of 0.45 units/hour, a sliding scale, and a carbs ratio of 1:20. His current glycemic regimen consisted of 15 IU of insulin aspart before meals and a sliding scale of insulin aspart, both delivered via an insulin pump. According to the patient, he had a new pump a month before admission, and the setting had been changed two days before the current presentation. He was then transferred to our facility for a higher level of care.

Upon arriving at our facility, the patient had already received fluid resuscitation, and his vital signs showed a temperature of 98.6°F, a heart rate of 96, a blood pressure of 122/76, and a respiratory rate of 16 breaths per minute. The rest of the examination was benign. Initial laboratory tests from an outside facility showed hyperglycemia with a blood glucose level of 729 mg/dL (normal range: 74-106 mg/dL), and a full laboratory workup (Table 1) revealed high anion gap metabolic acidosis, lactic acidosis, ketosis, leukocytosis, and acute kidney injury. Urinalysis was negative for pyuria, leukocyte esterase, and nitrates but showed glucose levels greater than 500 mg/dL and ketones greater than 60 mg/dL. The patient was diagnosed there with DKA and treated with a continuous insulin infusion, fluid resuscitation with normal saline, and electrolyte replacement.

**Table 1:** Laboratory results

Test	Reference Range	Day 1	Day 2	Day 3	Day 4
Procalcitonin	<0.10 ng/mL		13.70	10.40	5.97
C-reactive protein (CRP)	0.0-0.3 mg/dL		1.58	1.03	
Arterial pH	7.35-7.45	7.15	7.38		
Arterial partial pressure of CO2 (PCO2)	35-45 mmHg	12	30		
Arterial partial pressure of O2 (PO2)	80-100 mmHg	126	98		
Hemoglobin	13-18 g/dL	11.8	10.8	11.4	10.9
White blood cell count	4.50-10 k/cmm	16.9	12.64	9.52	5.94
Neutrophils	2.00-8.00 k/cmm	14	9.52	6.28	2.78
Platelet count	150-440 k/cmm	297	270	274	269
Sodium	135-145 mEq/L	124	138	131	133
Potassium	3.5-5.0 mEq/L	5.6	4.3	4.6	3.7
Chloride	98-108 mEq/L	89	108	102	102
Bicarbonate	22-29 mEq/L	15	19.6	21	27
Anion gap	5-14 mmol/L	15	12	8	4
Lactic acid	0.70-2.10 mmol/L		1.10		
Creatinine	0.70-1.40 mg/dL	1.74	1.53	1.17	1.0
Lipase	73-393 Units/L			56	
Amylase	25-115 Units/L			20	

On day two, the patient's blood glucose level was 159 mg/dL, and the anion gap was 12. Amylase level was normal, EKG showed normal sinus rhythm with no T wave changes, and troponin trends were unremarkable. A procalcitonin test was ordered since he presented with leukocytosis, and the results were elevated at 13.7 ng/mL, but the patient did not have a fever or chills. He was stable and did not show any signs of an ongoing infection. The viral panel, blood, and urine cultures were negative for infection. The patient was then transitioned to subcutaneous insulin with a basal rate of 10 IU insulin glargine in the morning and 5 IU at night, a low-dose sliding-scale insulin, and 3 IU insulin aspart before meals. On day three, the procalcitonin level was trending downward at 10.40 ng/mL. The patient was discharged to the general medical floor after his glycemic levels stabilized on this regimen.

## Discussion

Procalcitonin (PCT) has emerged as a helpful inflammatory marker in distinguishing bacterial sepsis from other causes of infection. However, they can also increase in conditions that elevate cytokine levels, like burns, chronic kidney disease, trauma, myocardial infarction, and stroke [4]. 

PCT is synthesized in the thyroid C cells and stored as a precursor until it is cleaved into calcitonin. Thus, in healthy subjects, PCT is undetectable [7]. However, during infection, the release of cytokines enhances the production of PCT from neuroendocrine cells from different organs such as the lungs, kidneys, liver, and intestine [8].

It is well-established that diabetic ketoacidosis (DKA) enhances leukocytosis and releases cytokines ([e.g., interleukin {IL}]-6, tumor necrosis factor (TNF)-alpha, and IL-1b) unrelated to infections [9,10]. Few studies have suggested that hyperglycemia is associated with the elevation of PCT, and the correction of glycemic levels demonstrated a lowering of PCT [5,6]. Another study by Ivaska et al. reported that PCT can be elevated in children with Type 1 DM with DKA despite the absence of infection [11]. There are few case reports to our knowledge that demonstrate the elevation of PCT in the setting of DKA in the absence of infection [4-6].

Our patient presented with evidence of diabetic ketoacidosis with nausea, vomiting, hyperglycemia, and a high anion gap. Procalcitonin was elevated, which led to more investigation to look for the source of the infection. All studies and cultures (blood and urine) were negative. After the treatment of DKA with hydration and insulin administration without antibiotics, his PCT decreased.

Thus, it is important to use biomarkers such as procalcitonin (PCT) judiciously when evaluating patients with DKA, especially when considering the use of antibiotics. While elevated levels of PCT can be indicative of a bacterial infection in many cases, it is important to consider other potential causes of elevated PCT levels, such as non-infectious inflammation in DKA.

To avoid the unnecessary use of antibiotics and minimize the cost of treatment, healthcare providers should carefully evaluate the patient's overall clinical picture and consider additional diagnostic tests to help distinguish between infectious and non-infectious causes of inflammation.

It is also important to consider the potential risks associated with the use of antibiotics, including the development of antibiotic resistance and adverse reactions to the medication. Antibiotics should only be used when necessary and appropriate, and healthcare providers should follow current guidelines for the treatment of DKA to ensure optimal management of the patient's condition.

## Conclusions

Appropriate use of these biomarkers, such as PCT, can help healthcare providers distinguish between infectious and non-infectious causes of inflammation. However, PCT has been observed to be elevated in DKA patients in the absence of infection. As with any biomarker, it is important to interpret PCT results in the context of the patient's overall clinical picture, taking into account other signs and symptoms of infection or inflammation as well as any underlying medical conditions that may be contributing to the patient's presentation.

Overall, a thorough and systematic approach to the evaluation and management of patients with DKA can help ensure optimal treatment and minimize the risk of unnecessary antibiotic use.
